# Graph spectral analysis of protein interaction network evolution

**DOI:** 10.1098/rsif.2012.0220

**Published:** 2012-05-02

**Authors:** Thomas Thorne, Michael P. H. Stumpf

**Affiliations:** Centre of Integrative Systems Biology and Bioinformatics, Division of Molecular Biosciences, Imperial College London, London SW7 2AZ, UK

**Keywords:** protein interaction networks, graph spectra, approximate Bayesian computation, network evolution, sequential Monte Carlo

## Abstract

We present an analysis of protein interaction network data via the comparison of models of network evolution to the observed data. We take a Bayesian approach and perform posterior density estimation using an approximate Bayesian computation with sequential Monte Carlo method. Our approach allows us to perform model selection over a selection of potential network growth models. The methodology we apply uses a distance defined in terms of graph spectra which captures the network data more naturally than previously used summary statistics such as the degree distribution. Furthermore, we include the effects of sampling into the analysis, to properly correct for the incompleteness of existing datasets, and have analysed the performance of our method under various degrees of sampling. We consider a number of models focusing not only on the biologically relevant class of duplication models, but also including models of scale-free network growth that have previously been claimed to describe such data. We find a preference for a duplication-divergence with linear preferential attachment model in the majority of the interaction datasets considered. We also illustrate how our method can be used to perform multi-model inference of network parameters to estimate properties of the full network from sampled data.

## 1. Introduction

Protein–protein interactions are one of the mechanisms by which biological organisms build complicated and flexible molecular machineries from relatively modest numbers of protein-coding genes. Similar to the way in which we can derive information about the evolution of genes and genomes through currently available high-throughput genome sequencing data, the availability of high-throughput protein interaction data from Yeast-2-Hybrid experiments and various other protocols gives us a snapshot of the evolutionary process by which the rich and complex structure of protein interactions in the cell is formed.

The nature of current protein interaction network (PIN) data presents challenges in analysing the data and performing inference that takes into account the global network structure. When considering evolutionary models we are faced with the problem of comparing the network structure produced by the model to that of the observed interaction network. A possible way of overcoming this problem is to calculate summary statistics describing some aspect of the data and compare these with predictions from evolutionary models. Several previous studies in the literature have applied summary statistics to compare the fit of network models to observed data [1–4], shedding some light on aspects of network evolution and organization. Early studies suggested that the scale-free (SF) network models [5] might fit the observed PIN data well [3,6], but there have since been several and statistically robust challenges to this claim [7–9].

Considering more realistic biologically grounded models of network evolution has provided insights into potential mechanisms of PIN formation, and provides more readily interpretable and applicable results than those found by considering more general random graph models; in particular, it has become apparent that it is important to consider models of network growth (instead of static random graph models) even though they are vastly oversimplified compared with the real process of network evolution. A number of models have been proposed and analysed with respect to the observed data [1,2,10–12], all with the same general mechanism of node duplication, corresponding to gene duplication and subsequent divergence in function and of interactions.

Assessing the fit of various network growth models to the *Drosophila melanogaster* protein interaction network, Middendorf *et al.* [13] found that a duplication model best describes the data. A similar result was found in Ratmann *et al.* [4], where combining several different network statistics to compare the fit of models with the *Treponema pallidum* PIN, a model combining duplication divergence scheme with linear preferential attachment (LPA) was found to best explain the data. Plausible models should therefore include aspects of duplication followed by the ability of interactions to diverge and change with time.

Comparing models of network evolution—even if they are (by design) vastly oversimplified compared with the true process—holds the promise of allowing us to weigh up the relative contributions of different processes. For example, we may assess the relative role that duplication of individual proteins might have played in the evolution of natural systems. Ultimately, we would like to understand different processes and their roles in network evolution in a way that mirrors what is possible for sequence-based comparative analyses. Here, too, models are oversimplified (even if less severely) but have allowed us to disentangle different aspects affecting sequence evolution (codon usage, secondary structure constraints, etc.). More immediately, however, such evolutionary models also allow us to apply the comparative method to networks more meaningfully than mere lists of network characteristics would be. Comparative biology predates the availability of sequence information, of course, and here we will discuss models of network evolution in a manner akin to that used in classical morphologically based comparative studies [14].

Evolutionary analysis at the level of network organization is fraught with considerable technical challenges: the data are often noisy and incomplete; networks are notoriously hard to describe in terms of summary statistics; and calibrating evolutionary models against the available data (or summary statistics) is also non-trivial. Here, we develop a flexible and robust inferential framework to deal with these three issues. Our approach is aimed at estimating the ‘effective’ parameters of models of network evolution against network data, and choosing between different plausible models of network evolution whenever possible. We employ a Bayesian framework that allows us to deal with different candidate models and the uncertainties and problems inherent to the PIN data; and we use concepts from spectral graph theory to describe the networks, rather than relying on summary statistics.

Because the likelihood of general network growth models is computationally difficult to evaluate, we adopt an approximate Bayesian computation (ABC) approach; in ABC procedures the data (or summary statistics thereof) of model simulations (with parameters, *θ*, drawn from the prior) are compared with the real data and if a suitable distance measure between the data/summary statistics falls below a tolerance level, *ε*, then *θ* is accepted as a draw from the (ABC) posterior distribution. If *ε* → 0, then the ABC posterior will be in agreement with the exact posterior, as long as the whole data are used. Use of summary statistics can be problematic for parameter inference and model selection if statistics are not sufficient. This is unlikely ever to be the case for networks and therefore the spectral perspective taken here, which captures the whole data, is particularly pertinent.

Below we outline the ABC framework employed here and its use in parameter estimation, model selection and model averaging contexts. After discussing the spectral graph measures, we outline different evolutionary models, and describe how we can analyse incomplete network datasets. We then illustrate our approach against simulated data before considering real protein–protein interaction data. We conclude with a discussion of the results and will make the case for the statistically informed analysis of such simple models in the context of evolutionary systems biology.

## 2. Methods

### 2.1. Approximate Bayesian computation and sequential Monte Carlo methods

Models of network evolution differ in their complexity and in the details of the evolutionary process that they capture. Statistical model selection techniques are therefore required in order to compare their relative ability to capture the observed network data and explain the underlying evolutionary mechanisms. In particular, such approaches allow us to strike a compromise between the complexity of a model, and its ability to describe observed data. Here, we adopt a Bayesian framework, which treats the problems of parameter estimation and model selection analogously and does not require the *post hoc* use of, for example, an information criterion (in fact, the popular Bayesian information criterion, BIC, is an approximation to the conventional Bayesian model selection framework).

Given an observed protein interaction dataset, *D*, and a set of models *m*_*i*_, *i* = 1, 2, …, *M*, the Bayesian approach requires us to calculate the posterior probability distributions of the different models and their respective parameter sets *θ*_*i*_. We hence seek to evaluate 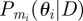, given by
2.1
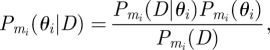

where 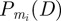 is the *evidence* for the data under model *m*_*i*_

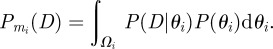



The complexity of the data and the models, however, makes evaluation of the likelihood terms, 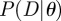, difficult and often impractical. To this end, ABC schemes have recently gained in popularity, especially in the fields of population, evolutionary and systems biology. In ABC frameworks, we forego evaluation of the likelihood in favour of comparing simulation outputs, 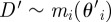 (for parameters sampled from the prior, 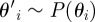), with the actual data, via a suitable distance measure, *d*(*D* ′, *D*). This allows us to approximate the posterior distribution as
2.2
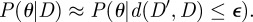

Here, it is important to note that the distance measure *d*(*D* ′, *D*) can also be applied to summary statistics of the data, *t*(*D*), rather than the actual data. This is especially attractive if the data are sufficiently complex such that the probability of observing the data is markedly reduced compared with observing the realized value of the summary statistic. But if the statistic is not sufficient (in the sense that 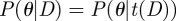), then parameter estimation and model selection become skewed compared with the full Bayesian approach.

Although it is possible to perform ABC using a simple rejection scheme, such a method will of course not be able to cope with models that have many parameters; however, several improved computational schemes exist and here we have chosen to apply the sequential Monte Carlo (SMC) method of Toni & Stumpf [15], which allows us to combine model selection and posterior density estimation in a single framework. SMC methods [16,17] operate on a population of weighted particles that correspond to points in the parameter space, with the particle weights set so that the empirical distribution of the weighted particles converges asymptotically to the desired target distribution as the number of particles *N* → *∞*. The basic ABC-SMC approach taken from Toni *et al.* [18] is outlined in algorithm 1. In brief, we proceed by constructing a set of intermediate distributions that start from the prior, *P*(*θ*_*i*_), and converge towards the (ABC) posterior, equation (2.2). Each intermediate distribution *P*_*t*_(*θ*) is characterized by a population of particles which fulfil the criterion
2.3
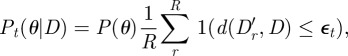

where *R* is the number of repeated simulations for fixed parameters and 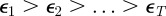 ensures that successive populations increasingly resemble the posterior (for which 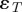 has to be sufficiently small).

This sequence is generated in practice through a sequential importance sampling procedure, which weights the different particles appropriately. To start with all particles are sampled independently from the prior, *P*(*θ*), and accepted or rejected according to whether simulated datasets agree with the observed data within the tolerance 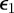, giving an initial set of *N* particles *θ*_1_^i^ for 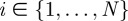.

In order to construct the next population of particles (for tolerance 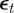), we have to propose new particles from the *θ*_*t*−1_^*i*^ making up population *t* − 1. To do so, we resample particles from the population at step *t* − 1 based on the particle weights, and then perturb these particles using a kernel in order to explore parameter space and reduce the degeneracy of the sample. Since our model parameters all take continuous values, we can construct our kernel by simply displacing particles by a distance drawn from a multivariate Gaussian distribution with zero mean and an appropriately selected variance to perturb the population of particles between successive iterations, so that
2.4
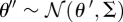

for some diagonal bandwidth matrix *Σ*, where *θ* ′ is a particle drawn from the present population, and *θ*″ is a new proposal. Other transition kernels are also possible, however.

Having selected and perturbed a particle to give us the proposed new parameters *θ*″ for the particle, the model is simulated with the new parameters to generate a test dataset *D*′, and the distance between this simulated data and the observed data *D* is calculated, using the distance measure *d*(*D*′, *D*) that we describe in §2.4. Then the proposed particle is accepted as being representative of the desired distribution only if it falls within a distance 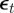 of the observed data and we can use equation (2.3) as an approximation of the likelihood,
2.5
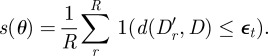



If *s*(*θ*) = 0, the particle is rejected and instead a new particle from the *θ*_*t*−1_^*i*^ is sampled and a new perturbation proposed.

To calculate the weight of the perturbed particle the method described in Del Moral *et al.* [17] is applied, whereby *w*_*t*_^*i*^ for the new parameters *θ*″ is calculated using our approximation of the likelihood *s*(*θ*) from equation (2.5) as
2.6
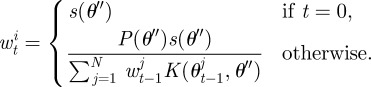



This is repeated until the desired number of particles have been sampled to give a new population *θ*_*t*_^*i*^ and the process is repeated using a progressively stricter sequence of distances 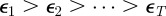 at each step. This procedure is outlined in algorithm 1.

### 2.2. ABC-SMC model selection

As mentioned previously, in order to include the different models under consideration into the inference procedure, we may simply treat models and parameters analogously and we can encode the choice of the model as a discrete parameter, following the methodology of Toni & Stumpf [15].

**Algorithm 1. Basic ABC-SMC algorithm.**

N ← Number of particles;

T ← Number of steps;

**for** t ← 1 **to** *T* **do**

 *i* ←1;

 **while** *i* < =*N* **do**

  **if** *t* = 1 **then**

   Sample *θ*″ ∼ *P*(*θ*);

  **else**

   Sample *θ*′ from *θ*_*t*−1_^*i*^ according to *w*_*t*−1_^*i*^;

   Perturb *θ*′ by *K*(*θ*′) to *θ* ″;

  **end**

  Simulate *D*′ from *θ* ″ *R* times;

  *s*(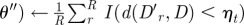;

  **if** *s* > 0 **then**

   
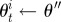

   **if** *t* = 1 **then**

    
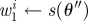

   **else**

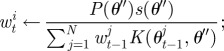

   **end**

   
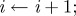

  **end**

 **end**

 Normalize *w*;

**end**

In such a context, model selection is then performed by considering the posterior density of each model marginalized over the parameters,
2.7
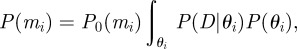

under some prior distribution *P*_0_(*m_i_*) over the models *m*_*i*_ ∈ ℳ. Taking this approach we can simply add an ordinal parameter indicating the model *m*^*j*^ of each particle *j* ∈ {1, …, *N*} used in the ABC-SMC algorithm, and doing so enables us to approximate equation (2.7), the marginal posterior probability distribution of the models for the population of particles at step *t*, as
2.8
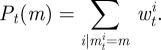



Then the procedure outlined in algorithm 1 is modified so that to generate a particle from population *t*, first a model is chosen according to its (marginal) probability, *P*_*t*−1_(*m*), before one of the corresponding particles is chosen. To perturb the resampled particle, two separate kernels, *K*_*M*_ and *K*_*θ*_, are used; the first is used to propose a new model and the second to perturb the model parameters. Here, for our kernel on the choice of model, we propose to move to a new model chosen uniformly at random with probability *p*, or to stay with the current model with probability 1 − *p*, although other choices of kernel are again possible. The update of the particle weights then also takes the model parameter into account,
2.9
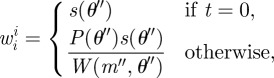

where
2.10
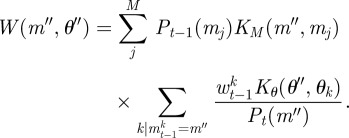



This procedure is outlined in algorithm 2, and described in more detail in Toni & Stumpf [15]. After performing the inference procedure, the final population of particles at step *T* can then be used with equation (2.8) to derive the posterior model probabilities.

### 2.3. Model averaging

In many circumstances, it is not possible to decide in favour of any particular model; in such cases, the posterior probability of several candidates is appreciable and comparable and analysis should proceed by pooling the results/predictions from these models, weighted by the relative evidence in their favour. This is precisely the aim of the Bayesian model averaging. As had previously been explored in Stumpf & Thorne [19], when fitting several different models to interaction network data, it is possible to improve the accuracy of predictions by averaging inferred statistics over all of the models [20].

**Algorithm 2. ABC-SMC model selection algorithm.**

*N* ← Number of particles;

*T* ← Number of steps;

**for** *t* ← 1 **to** *T* **do**

 *i* ← 1;

 **while** *i* < =*N* **do**

  **if** *t* = 1 **then**

   Sample *m*″ ∼ *P*_0_(*m*);

   Sample *θ*″ ∼ *P*_*m*_*i*__(*θ*);

  **else**

   Sample *m*′ ∼*P*_*t*−1_ (*m*);

   Sample *θ*′ from *θ*_*m*′,*t*−1_^*i*^ according to *w*_*m*′,*t*−1_^*i*^;

   Perturb *m*′ by *K*_*M*_(*m*) to *m*″;

   Perturb *θ*′ by *K*_*θ*_(*θ*) to *θ*″;

  **end**

  Simulate *D*′ from *m*″, *θ*″ *R* times;

  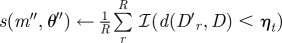;

  **if** *s* > 0 **then**

   *m*_*t*_^*i*^ ← *m*″;

   *θ*_*t*_^*i*^ ← *θ*″;

   **if** *t* = 1 **then**

    *w*_0_^*i*^ ← *s*(*m*″, *θ*″);

   **else**

    
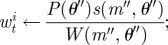

   **end**

   *i* ← *i* + 1;

  **end**

 **end**

 Normalize *w*;

**end**

Our method gives us the posterior probabilities for each model under consideration as
2.11
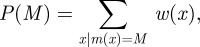

and so we can easily average an inferred parameter or statistic over all of the models by simply taking the weighted average of the value given by each model
2.12
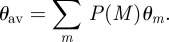

It has to be borne in mind, however, that the role of parameters (such as the rate of duplication) can differ quite considerably between models depending on the other factors considered by different models. Nevertheless even then predictions (e.g. for the total number of interactions in a network, or any aspects of the graph spectrum) can improve under such a model averaging scheme.

### 2.4. Network distance measure

Given a graph *G* comprising a set of nodes *N* and edges (*i*,*j*) ∈ *E* with *i*,*j* ∈ *N*, the adjacency matrix *A* of the graph is defined as the |*N*|·|*N*| matrix having entries
2.13
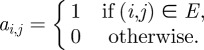

The adjacency matrix captures all aspects of the network structure and is therefore a complete representation of the observed data, rather than a summary statistic (such as degree distribution, clustering or centrality measures, motifs or graphlets). Here, we only consider undirected networks and so taking edges (*i*,*j*) as an unordered pair the adjacency matrix *A* will be a real symmetric matrix. Clearly, the structure of the adjacency matrix depends on some ordering of the nodes *N* and will not be unique for an unlabelled graph. Thus, isomorphic graphs may not necessarily have identical adjacency matrices, even though their network structures are the same. Of course, a simple relabelling of nodes will lead to identical adjacency matrices.

A simple distance measure between graphs having adjacency matrices *A* and *B*, known as the edit distance, is to enumerate the number of edges that are not shared by both graphs,
2.14
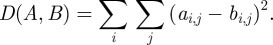



However, for unlabelled graphs, we are interested in some mapping *h* from *i* ∈ *N*_*A*_ to *i*′ ∈ *N*_*B*_ that minimizes the distance
2.15
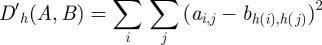

over all possible mappings of nodes between the two graphs, since there is no fixed correspondence between the unlabelled nodes. This mapping can be formulated by applying some permutation matrix *P* to the matrix *B*. Then we seek to evaluate *D* ′_*P*_ for *P* equal to the (unknown) optimal permutation matrix 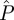, corresponding to the mapping that minimizes the distance *D* ′_*P*_,
2.16
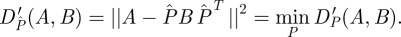



Since the evolutionary models we consider produce unlabelled graphs (although we could label them, any such labelling would necessarily be arbitrary and this would impose an undesirable loss of generality in the models), we require the latter form of the distance measure, 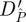.

Considering all possible permutations for pairs of networks of some 5000 nodes, such as the *Saccharomyces cerevisae* PIN, would be prohibitively expensive, but fortunately it is possible to approximate an optimal permutation. Considering the distance measure 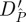 we can then apply the theorem of Umeyama [21], which gives us an approximate lower bound on the edit distance between two graphs as
2.17
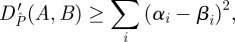

where it is assumed that both *A* and *B* are Hermitian matrices and *α*_*i*_ and *β*_*i*_ are the ordered eigenvalues of *A* and *B*. Although this distance measure only gives us a lower bound on the edit distance between the graphs, it has been shown in Wilson & Zhu [22] that this distance measure is an excellent approximation and accurately reflects the edit distance measure between two graphs. The distance given by equation (2.17) is an approximation of the distance between the complete data and not summary statistics of the network as had been used previously in composite likelihood [19] and ABC analyses [4] of networks.

The matrix eigenvalue calculation can itself be computationally expensive, and so in our implementation, we have used highly optimized commercial LAPACK routines running on GPGPU hardware that provides performance several orders of magnitude faster than a regular CPU for problems of the size we consider here.

### 2.5. Network growth models

Many random network models have been proposed in the literature, and here we have chosen models with a preference for those with some biological relevance, in the hope that they may help us to elucidate the processes of network evolution. It would entirely be possible to also consider static network models, such as Erdös–Rényi [23] or geometric graphs [24], but these provide no insights into the generative mechanisms underlying the evolution of biological networks.

We have chosen to take the prior probabilities *P*(*θ*) of the model parameters and the prior *P*(*m*) of the models themselves to be uniform over some appropriate range, since in the absence of any prior knowledge directly corresponding to the model parameters or a concrete preference for any particular model this seems to be the most parsimonious approach. Below, we will discuss the models in the necessary detail required to understand our results and discussion.

#### 2.5.1. Duplication models

**Figure 1.**
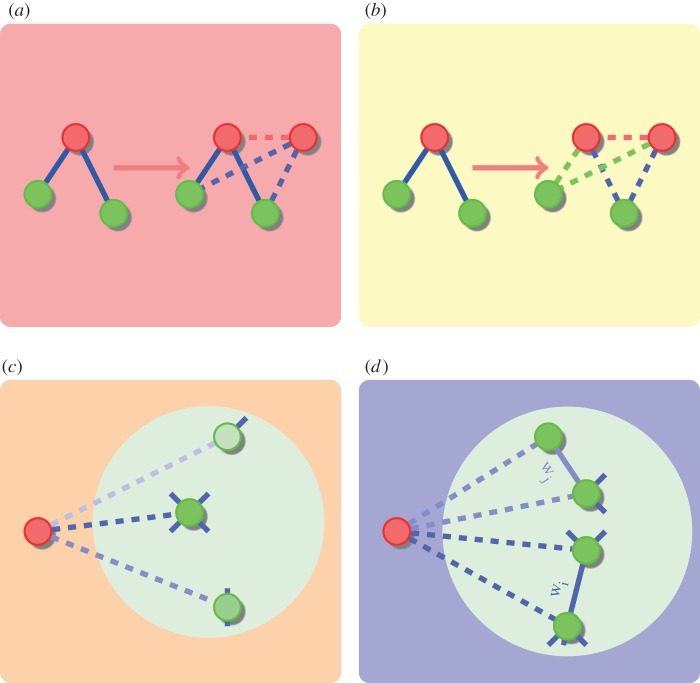
(*a*) Duplication attachment (DA) model. A node (red) is chosen to be duplicated and the duplicated node inherits the interactions of the original with probability 1 − *δ*, or diverges and loses the interaction with probability *δ*. An edge between the original and duplicated nodes is added with probability *α*, modelling the possibility of a self-interaction that is preserved. (*b*) Duplication attachment with complementarity (DAC) model. The model proceeds as the DA model, except that at least one edge in each of the green/blue pairs will be kept in the case of a divergence event, but either the interaction of the original or the duplicated node may be lost. (*c*) Linear preferential attachment (LPA) model [5]. At each time step, a new node is added to the network, and edges formed to the existing nodes with a probability proportional to their degree, so that edges are preferentially added to existing nodes of high degree. (*d*) General SF model [25]. The general SF model is a more sophisticated preferential attachment scheme whereby the scaling coefficient of the resulting degree distribution can be altered by the model parameters. Edges begin with weight 1, and as new nodes are added to the network at each time step, a random existing edge is chosen based on the edge weights, and a node at one end of the edge selected at random is chosen for the new node to be connected to. The edge weight of the chosen edge is then increased by the parameter *m*.

We have considered two different duplication divergence models based on those proposed in the literature. The simplest model we examine is the duplication–divergence–heterodimerization model [2,12], allowing for interactions to form between the original and the duplicated node, corresponding to heterodimerization. This model, which we will refer to as a duplication attachment (DA), illustrated in figure 1, selects a node uniformly at random from the network and duplicates the node, keeping each edge with some probability 1 − *δ* or diverging and losing the interaction with probability *δ*, always leaving he edges of the original node intact. Furthermore, an edge between the original and duplicated nodes is added with probability *α*, corresponding to the duplication of a heterodimer.

We also consider a DA preserving complementarity (DAC) model, similar in construction to those described in Ispolatov *et al.* [12] and Vazquez *et al.* [2], where the complementarity of edges is preserved, but allowing edges to be lost from both the original and duplicated node when divergence occurs, rather than only asymmetrically from the original node. As shown in figure 1, either the edge of the original node and its counterpart from the duplicated node are both kept with probability 1 − *δ*, or in the case that divergence occurs (with probability *δ*) one of the edges is selected at random and deleted, so that at least one of the pair is always kept. Again an edge is also added between the original and duplicated nodes with probability *α*.

#### 2.5.2. Scale-free models

A network is considered SF if the degree distribution of the nodes follows a power-law distribution in the limit of infinite network size, so that for node degrees *k*, *P*(*k*) ∼ *k*^−*a*^, for some scaling coefficient *a*. We consider both the LPA scheme of Barabasi & Albert [5] and the more complex scheme of Dorogovtsev & Mendes [25] that allows for the specification of the scaling coefficient as a model parameter.

The LPA model of Barabasi & Albert [5] grows the network by adding a single node at each time step, and attaching edges from this node to those in the existing network with a probability proportional to their degree, and is illustrated in figure 1. Thus for a node in the network of degree *k* the probability of attachment is *k*/2*M*, where *M* is the total number of edges in the network. We also allow for multiple edges to be added at each step by sampling the number of edges to add from a Poisson distribution with mean *m*. If we did not do so, then it would not be possible to grow networks with a ratio of nodes to edges other than 1:1; the Poisson distribution is a convenient way of ensuring that the number of nodes and edges in the real network can be achieved in the simulated data.

Such a scheme will produce a network with a degree distribution whose scaling coefficient is always 3 [5], whereas the generalized SF method of Dorogovtsev & Mendes [25] allows us to further parametrize the model to vary the scaling coefficient of the degree distribution. We omit the details but describe the model briefly below, and illustrate the growth step in figure 1. Edges in the network are assigned weights, all of which are initially set to 1. At each time step, a new node is added to the network, and again a number of edges sampled from Pois(*m*) is added from this node. Rather than adding edges preferentially based on the degree of nodes, the edge weights are used to select an edge, and the new node is attached to a randomly selected end of the chosen edge. Finally, the weight of the selected edge is increased by (the parameter) *ω*. Such a scheme generates a network with scaling coefficient 2 + (1/(1 + 2*ω*)) [25].

#### 2.5.3. Generalized models

We also consider two alternative models that allow for both duplication-divergence dynamics, as well as random addition of edges by either a uniform random attachment [26] or a preferential attachment scheme [5]. Both models employ a parameter *p*, the probability of performing a duplication move at each step, while a random edge addition move is performed with probability 1 − *p*. Again, we allow for multiple edges to be added during each step for the random edge addition moves, with the number of edges to be attached drawn from a Poisson distribution with parameter *m*.

The first such model combines the DA preserving complementarity scheme described above with a simple random addition of edges (DACR). During a random edge addition step, the new node is added to the network, and then a number of edges is sampled according to Pois(*m*), and each edge is assigned to two nodes chosen uniformly at random from the network.

The second model again uses the duplication divergence preserving complementarity scheme but uses LPA for the edge addition steps (DACL). Thus, during a random edge addition step the new node is added to the network, a number of edges is sampled according to Pois(*m*), and we attach each edge from the new node to the existing nodes with a probability proportional to their degree.

### 2.6. Sampling

It has previously been reported [19,27,28] that the effects of sampling on network data can bias inferences made under the assumption that the network structure of the subsample is representative of the structural properties of the full network. Since the network data we are using are in fact only a subnetwork of the interaction network existing in the organism, we include this fact in our model to prevent the effects of sampling from biasing the results. Currently available interaction datasets only include a subset of the genes known to exist in the respective organism, and so we apply a simple model of a sampling scheme to attempt to include the incompleteness of the data in the analysis. In the absence of more detailed information on the experimental sampling applied in generating the data, we take the parsimonious approach of assuming that each protein in the full interactome is sampled uniformly to yield the observed interaction data.

The sampling model is incorporated into our method by growing networks up to the size of the number of genes known to exist in the organism in question rather than the number of proteins in the interaction dataset. A random subset of the nodes of the network is then taken to reduce it in size to the same number of nodes as the observed interaction data and the subnetwork induced by these nodes is then used in the analysis in place of the larger network. While this methodology will allow for our induced subnetworks to include nodes of degree zero, which are absent in the observed data, in the absence of any tractable alternative methodology, we feel such an approximation is a suitable trade-off in allowing us to consider the effect of sampling on the inference. Thus, in our inference of degree distributions described in §3.2.2, we correct for the fact that the available data never contain nodes of degree zero.

In order to simulate network data for a particular model in the ABC-SMC algorithm, we apply the method outlined in algorithm 3. This gives us a network of the same number of nodes as the sampled interactome data being used, but allows us to infer the parameters of the full network by growing our simulated models to the size of the full proteome of the organism in question.

**Algorithm 3. Network growth model simulation with sampling.**

**Input**: model *m*, parameters *θ*, *N*_*T*_ proteins in organism, *N*_*S*_ proteins in interactome data of organism

**Output**: Sampled network of *N*_*S*_ nodes, grown from model *m* with parameters *θ*

Grow network *a* with *N*_*T*_ nodes, according to model *m* and parameters *θ*;

Create empty network *b*;

**for** *i* ← 1 **to** *N*_*S*_ **do**

 Sample node *x* from Nodes(*a*) \ Nodes(*b*);

 Add node *x* to *b*;

**end**

**for** (*x*,*y*) ∈ Edges(*a*) **do**

 **if** *x* ∈ Nodes(*b*) *and* *y* ∈ Nodes(*b*) **then**

  Add edge (*x*,*y*) to *b*;

 **end**

**end**

**return** *b*

### 2.7. Implementation

The method described was implemented in a mixture of Python (www.python.org) and C ++ code [29], with the framework of the SMC method implemented in Python, while using C ++ to improve the performance of the network growth model simulation code. Software is publicly available from www.theosysbio.org. As mentioned previously the matrix eigenvalue computations become prohibitively expensive for networks of the size considered here (e.g. around 5000 nodes). Therefore, we have used the CULAtools GPU linear algebra library (www.culatools.com) to perform the matrix calculations on GPGPU hardware, greatly increasing the speed of the calculations compared to a conventional CPU implementation. Using the CULAtools GPGPU LAPACK library implementation gives approximately a four times speed-up compared with a CPU optimized LAPACK implementation. Even with such optimizations producing posterior estimates can be costly, and takes around 12 h using an NVIDIA Tesla C2050 GPU and a 6 core 3.3 GHz Intel Core i7 CPU.

## 3. Results

Network evolution is a highly complex and contingent process; by design, the models considered here are vastly oversimplified compared with the true evolutionary process. Because of the correlated nature of the data it is not expected that we can always unambiguously identify the true data-generating process. To investigate and illustrate this point—generic to reverse engineering problems [30,31]—we first consider synthetic data before an analysis of real protein–protein interaction data.

### 3.1. Simulated data

To evaluate the ability of our method to effectively approximate the posterior distribution of the model parameters, we have performed two tests on simulated data generated from a known model. We assess the performance of the method in estimating the parameters of a single model, and in performing model selection. Since sampling may have an impact on the ability to infer the posterior, as the data are deteriorated, we also test the performance of our two test cases under varying degrees of sampling, discarding a fraction of nodes in the simulated network.

Our simulated data are taken from the DACR model, with parameters *δ* = 0.4, *α* = 0.25, *p* = 0.7 and *m* = 3, grown to a size of 5000 nodes and having 25 099 edges.

**Figure 2.**
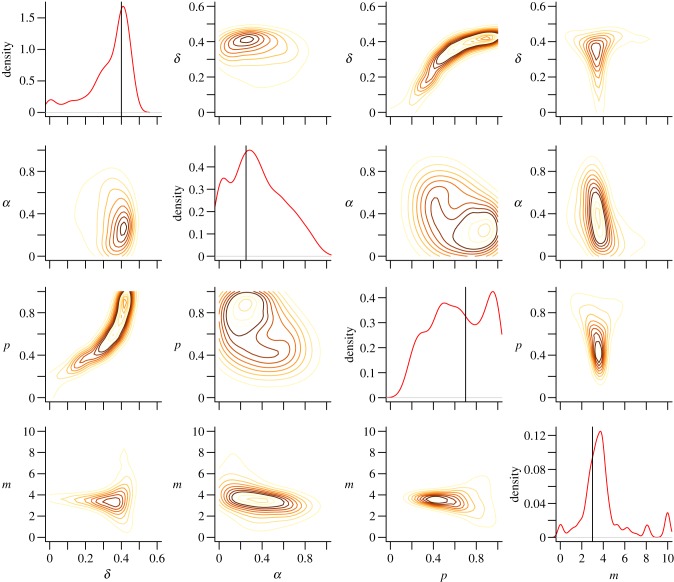
Posterior densities of each of the four parameters of the DACR model used to generate our test network. The actual parameter values are shown as vertical bars in the marginal density plots for each parameter. Contour plots illustrating the posterior densities of pairs of parameters are shown in the off-diagonal blocks.

Attempting to infer the known model parameters using the full dataset, we obtain the posterior densities shown in figure 2. It appears that the posterior probability is centred around the correct values, and considering the inference is attempting to infer parameters of a stochastic model from a single sample, uncertainty in the resulting posterior distribution is to be expected.

**Figure 3.**
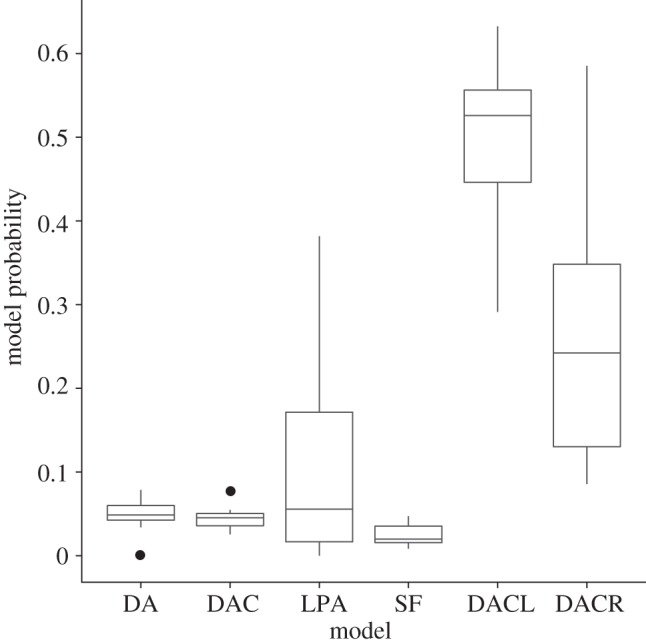
Distribution of posterior model probabilities for each of the models for our test data (generated from the DACR model) over 10 simulated datasets having identical parameters. While the posterior probabilities do not correctly identify the model used to generate the data as the most likely, there is a large variance in the results and this simply indicates that the data available are not sufficient to differentiate between the models.

**Figure 4.**
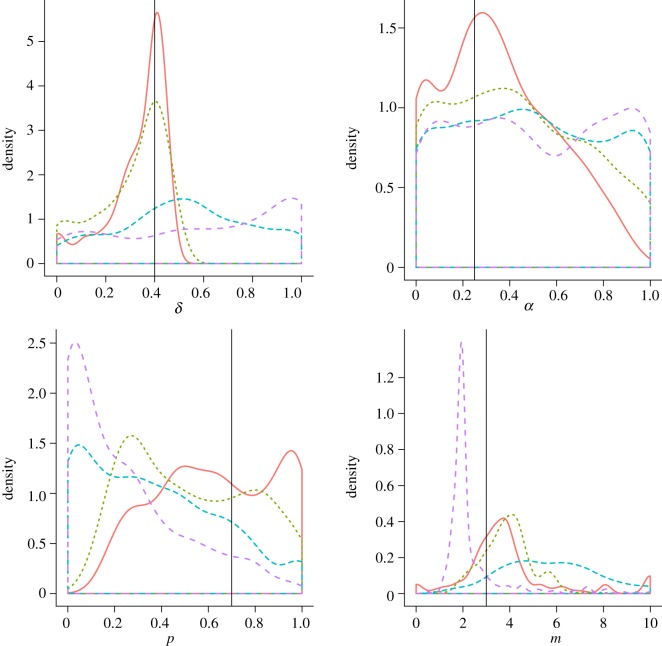
Posterior densities under different degrees of sampling for the four parameters of the DACR model used to generate the test network and samples. Sampled networks were generated by uniformly sampling a fraction of nodes and taking the induced subnetwork, for sample sizes of 75%, 50% and 25%. For samples of less than 50% of the nodes the posterior densities clearly differ from the actual model parameters (vertical lines). Samples: orange solid line, full; green dashed line, 75%; blue dashed line, 50%; purple dashed line, 25%.

The posterior model probabilities illustrated in figure 3 show that while the model from which the data were generated does not have the highest probability on average, it is still the second highest and the distributions of the values are close to overlapping, while the similar DACL model has the highest probability, suggesting that the single sample of network structure from the model is not sufficient to correctly discriminate the two. Taking samples from the generated data of 25, 50 and 75 per cent of the nodes, the posterior densities for the DACR model from which the data were generated shown in figure 4 reveal an interesting trend as the sampling fraction decreases. For the 75 per cent sample, the posterior distribution appears to be mostly centred around the same values as for the full network and almost as specific, whereas for the 50 and 25 per cent samples, the posterior distributions become much broader (and potentially biased), and particularly in the case of *α*, less specific and spread across the parameter range.

These findings reflect the general problems encountered in addressing the so-called inverse problems, and are not specific to the approach developed here. The consistency of statistical estimators is only an asymptotic property and for small data samples (here, we have only a single network) inferences are always subject to the variabilities of the data-generating process and the estimator. This explains also the need to consider Bayesian model averaging approaches.

### 3.2. Protein interaction network data

We applied our method to publicly available PIN datasets of varying completeness and size to allow us to examine the results and performance of the inference on differing kinds of data. The data used are summarized in table 1, and were downloaded from the Database of Interacting Proteins [32] (DIP; http://dip.doe-mbi.ucla.edu/dip/). The *S. cerevisae* dataset is the most complete, with a large fraction of the genes in the organism being included in the network, and a large number of edges. The *D. melanogaster* dataset is of a larger size, but represents a smaller fraction of the genes known to exist in the organism, while the *Helicobacter pylori* and *Escherichia coli* datasets are much smaller, and again represent small sampling fractions of their respective PINs.

**Table 1.** Summary of the PIN data used in the study. Datasets of varying size and sampling fraction were chosen so as to allow us to evaluate the performance of the method on a selection of different kinds of protein interaction data, representative of those currently available.

species	proteins	interactions	genome size	sampling fraction
*S. cerevisae*	5035	22118	6532	0.77
*D. melanogaster*	7506	22871	14076	0.53
*H. pylori*	715	1423	1589	0.45
*E. coli*	1888	7008	5416	0.35

#### 3.2.1. Model selection and model parameters

**Figure 5.**
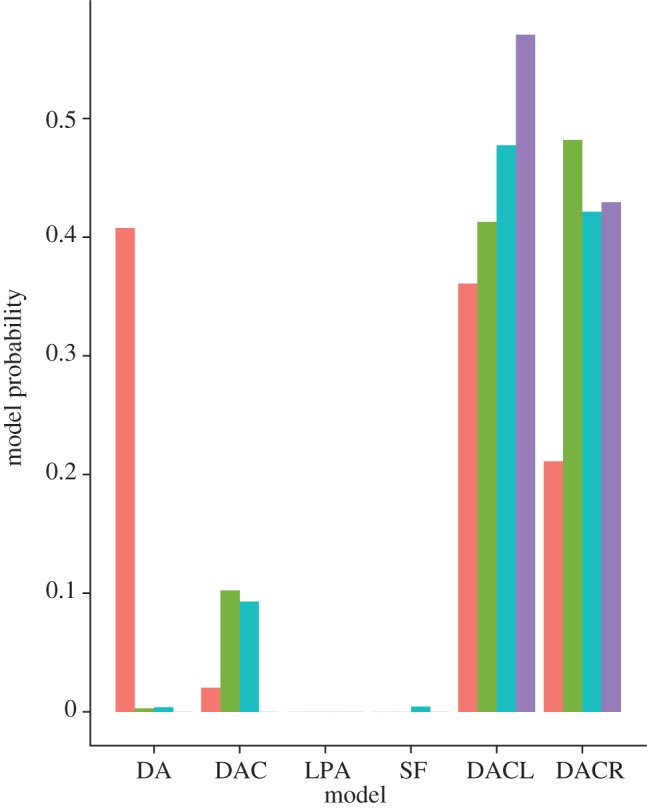
Model probabilities for each of the six models in the four species. There is a strong preference for the DACL or DACR models in all cases but for the *S. cerevisae* dataset, which is fit best by the DA model. Both of the SF models have near zero posterior probabilities, suggesting that they provide a poor fit to the data (orange, *S. cerevisae*; green, *D. melanogaster*; blue, *H. pylori*; purple, *E. coli*).

Applying our method to the protein interaction data summarized in table 1 we obtained the posterior model probabilities shown in figure 5. The results show a strong preference for the DACL and DACR models in almost all cases, except for in *S. cerevisae* where the largest posterior model probability corresponds to the DA model. Interestingly, in all cases the LPA and SF models have near zero probabilities, suggesting that these models do not fit the data as well as previously claimed. The majority of differences between the species appear to be between *S. cerevisae* and the other three species, with *D. melanogaster*, *H. pylori* and *E. coli* all exhibiting similar profiles. Interestingly, both *D. melanogaster* and *H. pylori* have a small probability for the DAC model not seen in the other species, while *E. coli* has a larger preference for the DACL model, and less so for the DAC model.

**Figure 6.**
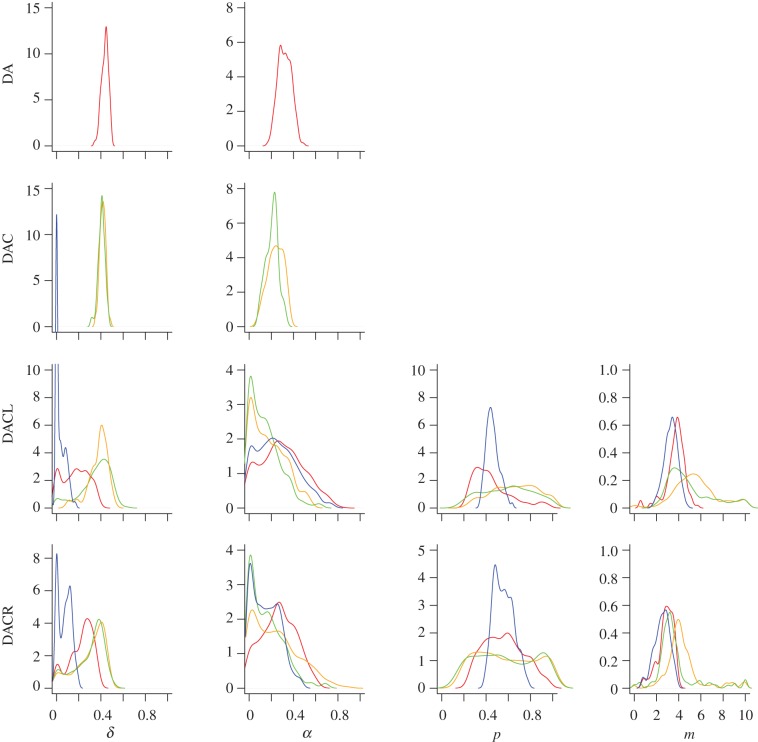
Posterior densities for the model parameters where there were a suitable number of particles in the posterior sample to calculate the distribution. Only the DA, DAC, DACL and DACR models had sufficient numbers of particles. The datasets are: red, *S. cerevisae*; orange, *D. melanogaster*; green, *H. pylori*; blue, *E. coli*.

Looking at the posterior density plots for the different species for the models where there were enough particles available to calculate the densities shown in figure 6, we see that the difference between the datasets is more clear. The most striking aspects are the similarity of the posterior densities for the *D. melanogaster* and *H. pylori* data across all of the models, and the significantly different shape of the posterior in *E. coli* for many of the parameters.

In many cases the posterior densities of the common parameters appear to be centred around similar values across all of the models, for example, with the parameter *δ*, common to all of the duplication models, the peaks are close and of a similar shape in most cases for the *S. cerevisae*, *H. pylori* and *D. melanogaster* data.

#### 3.2.2. Model averaging of network statistics

To evaluate the performance of our model averaging and sampling scheme, we attempted to infer the degree distribution of the observed *S. cerevisae* PIN data from our posterior particles for samples of 25 and 50 per cent of the nodes of the *S. cerevisae* PIN, as well as the full data.

**Figure 7.**
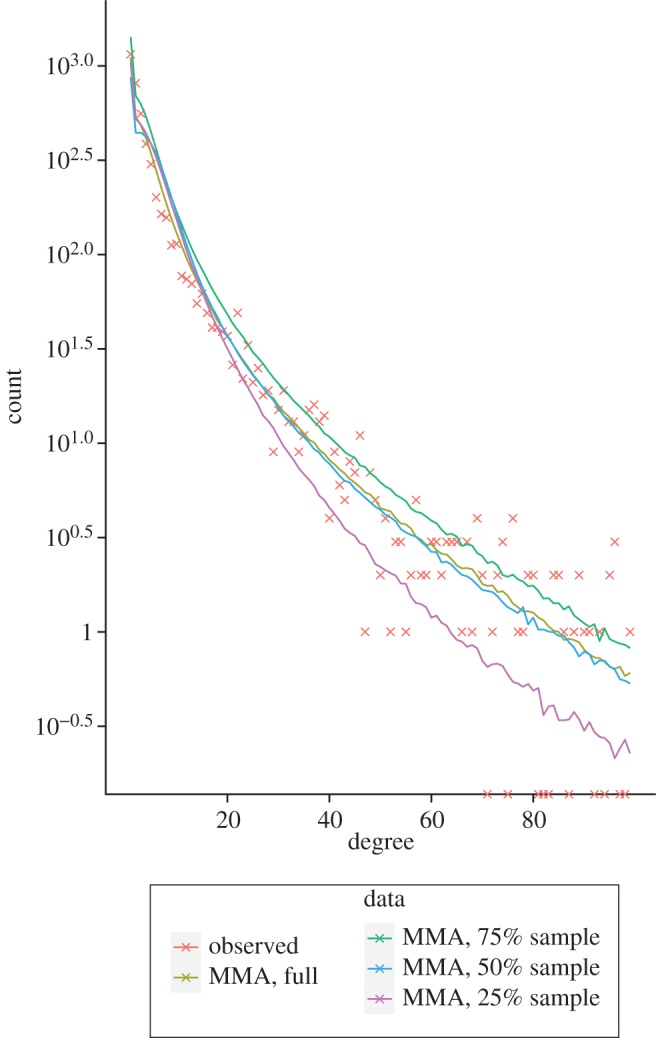
Degree distribution of the observed *S. cerevisae* PIN (orange), with plots of the model averaged distribution inferred from the posterior particles across all the models, using the full *S. cerevisae* dataset (olive), a 75% sample (green), a 50% sample (blue) and a 25% sample (purple).

As can be seen in figure 7, both the degree distribution inferred from the full *S. cerevisae* network and the 50 per cent sample appear to fit the data well, while the 25 per cent sample does not perform as well.

## 4. Discussion

The performance of our method on the generated test data (figure 2) illustrates the efficacy of our approach in reconstructing the model parameters for a given evolutionary model. While it would be unrealistic to assume our models correspond to the only mechanisms of network evolution at work and completely capture their behaviour, comparing such general mechanisms allows us to distinguish between the probable evolutionary processes at work. Although the model selection results in figure 3 show that we do not give the highest posterior probability to the correct model, the difference in mechanisms between the two generalized duplication models (DACL and DACR) is subtle and we correctly assign much lower probabilities to the other models, so this simply suggests that it may not be possible to tell them apart from a single network structure sampled from the stochastic DACR model.

As shown in figure 4, the effects of our sampling on the inferred parameters for our test dataset show that while the accuracy is reduced, we can in most cases reconstruct network parameters of the full network accurately for samples of 75 per cent of the full network. As the sampling size is reduced to 25 per cent the posterior distribution for the parameter *α* becomes spread across the parameter range, reflecting the inadequacy of the sampled data to allow for inference of the true parameter value. For the other parameters the results appear to be biased, suggesting that the sampling affects the ability of our inference procedure to infer each of the parameters in different ways.

The results of the model selection for the four PIN datasets we considered (figure 5) show that both of the SF models are poor at explaining the observed data in all cases. In agreement with the previous analysis of Ratmann *et al.* [4] and Middendorf *et al.* [13], the models combining duplication mechanics with some random addition of edges (DACL, DACR) seem to provide the best fit to the data, with only *S. cerevisae* showing a slight preference for the simplest DA model.

The posterior probabilities we infer for the model parameters of the different PIN datasets in figure 6 show an interesting pattern whereby the majority of parameters show similar distributions across the species, as well as across the different models. For example, the divergence parameter *δ*, describing the probability of duplicated edges being lost, appears to share a common value of around 0.4 across all of the models and the majority of the species, and the parameter *α* shows a similar trend although the posterior distributions are less specific for the DACL and DACR models. This may be due to the fact that inference of *α* appears to become increasingly difficult as the sampling fraction decreases, and three of our four PIN datasets represent small sampling fractions of around 50 per cent and less. The parameter *p* describing the probability of performing a duplication step or a random edge addition step appears to be unspecific except for in the case of *E. coli* where the posterior density seems to be centred around 0.5, while the number of edges added in each random edge addition step is around 3 for all the species.

The agreement between the posterior densities of *H. pylori* and *D. melanogaster* is striking especially due to the extreme difference in the size and the number of interactions of the datasets, with the *H. pylori* PIN being of a much smaller size. The *E. coli* dataset has a similarly small number of nodes as the *H. pylori* data, but far more edges relative to the number of nodes than any other datasets. This may go some way in explaining the differences apparent between the posterior parameter distributions for *E. coli* and the other species, or it may, on the other hand, be due to the low sampling fraction of the data, most probably a combination of the two.

Looking at the degree distributions, we infer for the *S. cerevisae* PIN by applying model averaging to the posterior particles inferred based on some sampled subsets of the observed network in figure 7, it appears that we can accurately reconstruct the degree distribution of the observed network based only on a sample of 50 per cent of the nodes. Then applying this to the four PIN datasets we have considered, we would expect that the *S. cerevisae*, *D. melanogaster* and *H. pylori* data would allow us to accurately reconstruct the degree distributions of the full networks from the sampled PIN data we have used. These results demonstrate the utility of our methodology not only in elucidating the evolutionary processes at work, but also in inferring properties of the as yet unknown full network structure from the sampled data by including the sampling process in our models.

## 5. Conclusion

We have demonstrated the ability of our method to correctly infer network growth model parameters from observed network data and illustrated its application to existing PIN data. We feel that our method provides both novel techniques and results that reveal insights into the evolutionary processes at work. As more complete and accurate protein interaction data become available in the future, we would expect these techniques to allow us to make progressively more precise predictions and comparisons between species.

Simple models like the ones considered here are vastly idealized and oversimplified models of a much more complicated and contingent evolutionary process. On the one hand, we use such models to gain qualitative insights into the evolution of networks; some of our models are somewhat more realistic compared with, for example, simple SF models, in the same sense in which Kimura's 2-parameter (K2P) model for nucleotide substitutions is arguably more realistic than the simpler Jukes–Cantor (JC) model. But neither the JC or the K2P, nor our models of network evolution consider functional, structural or indeed any biological constraints on the evolutionary dynamics. This may seem like a glaring omission, but is done out of necessity. First, we have no way of capturing these factors reliably and without extraneous and difficult to justify assumptions; second, these functionally ‘ignorant’ models can be used as null models/hypotheses. Comparing and contrasting real networks with those generated by simplified simulation models can highlight systematic differences; these can be caused by functional factors or by events such as whole genome duplications which are not captured by these evolutionary models.

A slightly more pragmatic use of such models and model calibration is for predictive purposes and comparison of large-scale network features between species. Bayesian model averaging has been shown to possess considerable predictive power even if the underlying models are known to be oversimplified or inadequate. Pooling over predictions weighted by the model fit to the data has the potential to yield testable and non-trivial predictions of the properties of complete networks (based on incomplete data).

An advantage of our approach is that spectral approaches allow us to compare networks more comprehensively than has previously been the case [26,33,34]. They incorporate implicitly the information contained in standard network summary statistics—degree distribution, clustering coefficient, distances, etc.—and also allow us a direct means of comparing graphs (in an ABC framework) rather than resorting to the more coarse-grained summary statistics that had been considered in the past [35]. As exact likelihood-based inferences are only possible for very simple growth models [36], the use of ABC is not only justified but also in fact unavoidable in model-based analysis of network evolution [4].

The statistical tools introduced here allow us to compare network data and models of their evolutionary dynamics; in principle, we can also choose to focus on either quantitative or qualitative aspects of network data, depending on the quality of available data (or the details captured by different models). Ultimately, there is no reason to be disappointed if all that we achieve is to reveal the inadequacies of existing models of network evolution. Being able to do so will in itself yield new insights into the evolutionary history of these networks.
